# S100B predicts neurological injury and 30-day mortality following surgery for acute type A aortic dissection: an observational cohort study

**DOI:** 10.1186/s13019-023-02151-2

**Published:** 2023-02-06

**Authors:** Karl Teurneau-Hermansson, Jacob Ede, Mårten Larsson, Marion Moseby-Knappe, Henrik Bjursten, Shahab Nozohoor, Johan Sjögren, Igor Zindovic

**Affiliations:** 1 Department of Clinical Sciences Lund, Department of Cardiothoracic Surgery, Lund University, Skåne University Hospital, 221 85 Lund, Sweden; 2 Department of Clinical Sciences Lund, Neurology, Lund University, Skåne University Hospital, Lund, Sweden

**Keywords:** Aorta, Dissection, S100B, Neurological injury

## Abstract

**Background:**

Neurological injuries are frequent following Acute Type A Aortic Dissection (ATAAD) repair occurring in 4–30% of all patients. Our objective was to study whether S100B can predict neurological injury following ATAAD repair.

**Methods:**

This was a single-center, retrospective, observational study. The study included all patients that underwent ATAAD repair at our institution between Jan 1998 and Dec 2021 and had recorded S100B-values. The primary outcome measure was neurological injury, defined as focal neurological deficit or coma diagnosed by clinical assessment with or without radiological confirmation and with a symptom duration of more than 24 h. Secondary outcome measure was 30-day mortality.

**Results:**

538 patients underwent surgery during the study period and 393 patients, had recorded S100B-values. The patients had a mean age of 64.4 ± 11.1 years and 34% were female. Receiver operating characteristic curve for S100B 24 h postoperatively yielded area under the curve 0.687 (95% CI 0.615–0.759) and best Youden’s index corresponded to S100B 0.225 which gave a sensitivity of 60% and specificity of 75%. Multivariable logistic regression identified S100B ≥ 0.23 μg/l at 24 h as an independent predictor for neurological injury (OR 4.71, 95% CI 2.59–8.57; *p* < 0.01) along with preoperative cerebral malperfusion (OR 4.23, 95% CI 2.03–8.84; *p* < 0.01) as well as an independent predictor for 30-day mortality (OR 4.57, 95% CI 1.18–11.70; *p* < 0.01).

**Conclusions:**

We demonstrated that S100B, 24 h after surgery is a strong independent predictor for neurological injury and 30-day mortality after ATAAD repair.

*Trial registration*: As this was a retrospective observational study it was not registered.

**Supplementary Information:**

The online version contains supplementary material available at 10.1186/s13019-023-02151-2.

## Background

Acute type A aortic dissection (ATAAD) is a potentially lethal condition, [1] and despite immediate surgical repair, ATAAD is associated with significant morbidity and mortality [2–7]. The incidence of ATAAD has been reported to be approximately 2 -16 cases per 100,000 persons/year [8, 9].

In patients who survive ATAAD and associated surgery, neurological injuries occur in 4–30% of patients [4, 10–15]. Both the aortic dissection itself and the surgical techniques used for ATAAD repair may contribute to the development of ischemic cerebral injury [5]. For instance, patients may present with cerebral malperfusion caused by compromised circulation or obstruction/occlusion of aortic branch vessels [10]. In addition, there are different surgical strategies for ATAAD repair such as site of arterial cannulation, usage of circulatory arrest and/or selective cerebral perfusion, and the degree of hypothermia, which all may have an impact on postoperative neurological outcomes [5, 6].

Patients with postoperative neurological injuries may benefit from neuroprotective management in the postoperative period, but to accurately target these patients, a reliable tool for early detection of neurological injury is needed, especially in sedated patients for whom routine clinical neurological assessment is not possible [16, 17].

Previous studies have shown the astrocytic marker S100B to be a valid marker of ischemic stroke and traumatic brain injury in the emergency setting [18, 19].

In routine cardiac surgery, postoperative elevated levels of S100B have been shown to correlate with ischemic stroke and other neurocognitive disorders, and S100B levels have been shown to correlate with the extent of neurological damage [20–23]. However, S100B and its usefulness as a predictor of neurological injury has not been assessed in the complex setting of ATAAD surgery, where preoperative cerebral malperfusion may be present and hypothermia and circulatory arrest are routinely used.

Therefore, the objective of this study was to study whether S100B can be used to predict neurological injury following ATAAD repair.

## Methods

### Study design

This was a single-center, retrospective, observational study performed at Skåne University Hospital which is a tertiary referral center with a catchment area of 1.9 million inhabitants. Between Jan 1998 and Dec 2021, 538 patients underwent surgical repair for ATAAD, and this study included all patients with recorded S100B values (*n* = 393). Data were prospectively entered into our departmental surgical database with missing values and additional variables collected by retrospective chart review. An aortic dissection was regarded as acute if the time from symptom onset to surgery was < 14 days. Ethical approval of this study was granted by the Swedish Ethical Review Agency (ref: 2021–01,185, date: April 23rd, 2021).

### Outcomes and definitions

The primary outcome measure was neurological injury, defined as focal neurological deficit or coma diagnosed by clinical assessment by a neurologist with or without radiological confirmation by computed tomography or magnetic resonance imaging with a symptom duration of more than 24 h. Secondary outcome measure was 30-day mortality.

Transient cerebral events (transient ischemic attack or transient stroke) were defined as neurological deficits with symptom duration of less than 24 h diagnosed by clinical assessment regardless of radiological findings. Hypotensive shock was defined as a preoperative period of systolic blood pressure < 90 mmHg, and preoperative cerebral malperfusion was defined as impaired consciousness or presence of clinical focal neurological symptoms before surgery. Postoperative stroke was defined as focal neurological symptoms persisting for more than 24 h postoperatively, while postoperative coma was defined as a state of unconsciousness (Glasgow Coma Scale Motor Score < 6) persisting more than 48 h without the influence of anesthetic agents.

### Sample collection

The blood samples were collected during routine lab monitoring using a central venous line at the following time points: T_0_ -preoperatively at arrival to the operating theatre; T_1_—within the first 12 h after surgery; T_2_—24 h after surgery; T_3_—48 h after surgery; and T_4_—72 h after surgery. S100B was analyzed by a monoclonal sandwich immune assay primarily using the Cobas 6000/8000 analyzer (Roche) but also using the Sangtec 100 analyzer (LIAISON) and Hitachi Modular-E analyzer (Roche).

### Surgical technique

The surgical technique used for ATAAD repair at our institution has been presented previously [24]. In brief, general anesthesia was routinely induced with propofol, fentanyl, and rocuronium bromide and maintained with propofol and remifentanil. Surgery was performed with median sternotomy, cardiopulmonary bypass, and intermittent cold blood cardioplegic cardiac arrest but specific surgical techniques were left to the discretion of each responsible surgeon. Arterial cannulation was commonly performed in the femoral artery or direct aortic cannulation aided by guidewire and transesophageal echocardiography, ensuring access to the true lumen. Bicaval cannulation or two-stage atrial cannulation was used for venous access. Distal repair was usually performed under hypothermic circulatory arrest (HCA). In some cases, antegrade or retrograde cerebral perfusion was employed. Before the circulation was stopped, our neuroprotective strategy included topical cooling of the head and administration of thiopental (1 g) and hydrocortisone (500 mg). After circulatory arrest, perfusion was restarted using a side-branch of the vascular prosthesis when the patient was in a Trendelenburg position to enable de-airing before clamping the vascular graft. Suturing of the proximal anastomosis and root and aortic valve procedures were performed during rewarming. An aortic root replacement was performed when the coronary ostia or aortic valve was affected by the ATAAD or in the presence of an aortic root aneurysm. Surgery on the aortic arch was performed in selected cases with an entry tear within the aortic arch, involvement of supra-aortic branch vessels, significant aortic arch dilation, or distal malperfusion. Other concomitant procedures were performed when necessary.

### Statistical analysis

Categorical variables were presented as numbers and percentages. Continuous variables were reported as median with interquartile range (IQR) or mean value ± standard deviation (SD) depending on the distribution of data. Chi-square test, Fisher’s exact test, two sample T-tests and Mann–Whitney U-test were used for intergroup comparisons when appropriate. Receiver Operating Characteristic (ROC) curves calculating the area under the curve (AUC) with a 95% confidence interval (CI) at each given time point were used to define a cutoff value for the best possible performance of S100B as a predictor of neurological injury at the earliest possible time point. The cutoff yielding the most favorable Youden’s index was then used as our grouping variable for postoperative outcomes. Uni- and multivariable logistic regression was used for identifying independent predictors of neurological injury and 30-day mortality. A *p*-value of 0.2 or less was used as the inclusion criterion for the multivariable model.

Multicollinearity between continuous variables was assessed by linear regression generating a Variance Inflation Factor (VIF). Spearman correlation was used for testing multicollinearity between categorical values and Pearson correlation between categorical and continuous values. Two variables with a VIF > 3.0 or correlation coefficient of > 0.5 were defined as being colinear. The goodness of fit of the multivariable models was assessed using the Omnibus Tests for Model Coefficients and the Hosmer–Lemeshow goodness of fit. A core multivariable model using the best possible cut-off was generated to determine independent predictors of neurological injury, but to generate an odds ratio for S100B as a continuous variable, an additional analysis was performed where the optimal cut-off for S100B was replaced by S100B as a continuous variable. In the multivariable analysis, the adjusted *p*-values were generated using the Bonferroni correction for multiple testing. For all tests, *p-*values < 0.05 were considered statistically significant. When calculating differences between groups missing values were excluded and the multivariable logistic regression relied on complete cases analyses. All statistical analyses were conducted using IBM^®^ SPSS^®^ Statistics version 27.0.1.0 for MacOS^®^ (IBM Corp, Armonk, NY, USA).

## Results

**Fig. 1:**
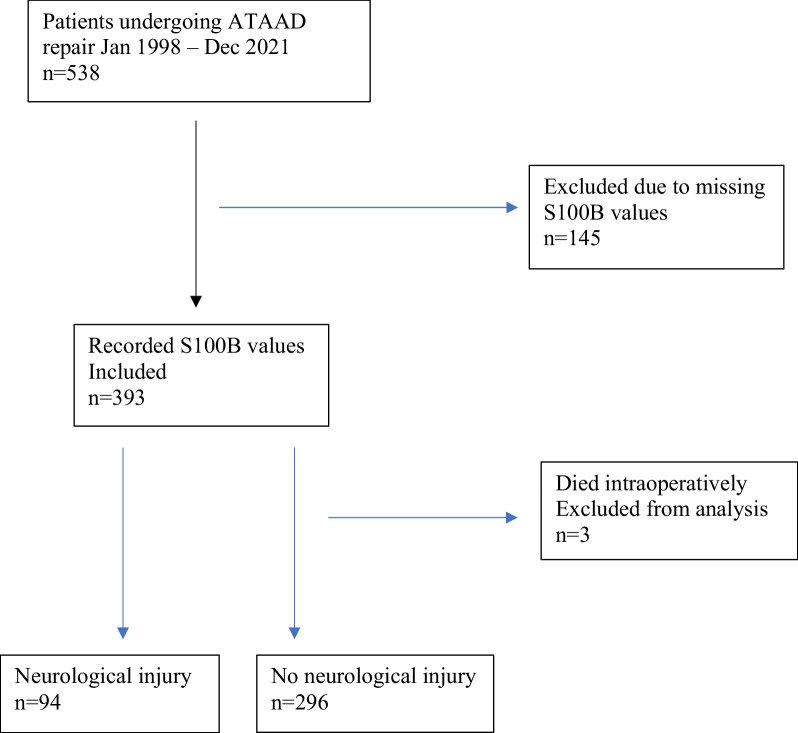
Flowchart of patient inclusion and exclusion

Between Jan 1998 and Dec 2021, a total of 538 patients underwent surgery for ATAAD at the Department of Cardiothoracic Surgery, Skåne University Hospital. Of these patients, 393 had recorded S100B-values and constituted our study cohort. Of the included patients, 94/393 (23.9%) suffered neurological injury as compared to 27/146 (18.5%) of the excluded patients (*p* = 0.16). Three patients who had recorded preoperative S100B-values but died intraoperatively were excluded resulting in S100B being available for 60 patients at T_0_, 299 patients at T_1_, 312 patients at T_2_, 183 patients at T_3,_ and 76 patients at T_4_. Figure 1 shows a flowchart of inclusion and exclusion.

The baseline variables of the study populations are presented in Table 1. The patients had a mean age of 64.4 ± 11.1 years, and 34% were female. There were no significant differences in age or gender distribution between the neurological injury and no neurological injury groups. Neurological injury was the more common group in patients with preoperative cerebral malperfusion (29% vs. 11% (*p* < 0.01)), other malperfusion (48% vs. 32% (*p* < 0.01)), and cardiac tamponade (21% vs. 12% (*p* = 0.04)). Preoperative creatinine and lactate levels were significantly higher in the neurological injury group compared to the no neurological injury group, but there was no significant difference in preoperative S100B levels. Diabetes was more common in the group that suffered neurological injury (29% vs. 18% (*p* = 0.02)).

**Table 1:** Baseline characteristics of the study population

Baseline data	All patients *n* = 393	Neurological Injury * n = 94	No Neurological Injury * n = 296	*p*	Missing
Age (years)	64.4 ± 11.1	65.5 ± 10.1	64.0 ± 11.5	0.26	0 (0)
Female	135 (34.4)	28 (29.8)	107 (36.1)	0.26	0 (0)
Hypertension	208 (52.9)	54 (57.4)	152 (51.4)	0.30	0 (0)
Diabetes Mellitus	80 (20.4)	27 (28.7)	52 (17.6)	0.02	0 (0)
COPD	26 (6.6)	4 (4.3)	21 (7.1)	0.33	0 (0)
Smoking	133 (35.5)	35 (40.2)	97 (34.0)	0.29	18 (4.6)
Coronary artery disease	24 (7.0)	5 (5.8)	19 (7.5)	0.60	50 (12.7)
Known thoracic aneurysm	37 (10.8)	7 (8.2)	30 (11.8)	0.36	51 (13.0)
Marfan syndrome	22 (5.6)	4 (4.3)	18 (6.1)	0.50	0 (0)
Other connective tissue disease	5 (1.3)	1 (1.2)	4 (1.6)	1.00	50 (12.7)
Family history of dissection	16 (4.1)	3 (3.2)	13 (4.4)	0.77	0 (0)
Previous cardiac surgery	7 (1.8)	2 (2.1)	5 (1.7)	0.68	1 (0.3)
Previous Aortic Surgery	12 (3.1)	2 (2.1)	10 (3.4)	0.74	1 (0.3)
Syncope	61 (17.8)	21 (24.4)	39 (15.4)	0.06	50 (12.7)
Hypotensive shock	81 (24.0)	26 (30.6)	54 (21.6)	0.01	55 (14.0)
Preoperative cardiac arrest	16 (4.7)	6 (7.0)	10 (3.9)	0.25	50 (12.7)
Cardiac tamponade	49 (14.3)	18 (20.9)	30 (11.8)	0.04	50 (12.7)
Any malperfusion	142 (36.1)	45 (47.9)	95 (32.1)	< 0.01	0 (0)
Cerebral malperfusion	58 (14.8)	27 (28.7)	31 (10.5)	< 0.01	1 (0.3)
*Carotid Dissection*				0.26	26 (6.6)
None Unilateral Bilateral	185 (50.4) 73 (19.9) 109 (29.7)	38 (42.7) 21 (23.6) 30 (33.7)	145 (52.7) 52 (18.9) 78 (28.4)		
Intramural hematoma	51 (14.9)	10 (11.6)	41 (16.2)	0.31	51 (13.0)
Debakey type 1	300 (76.7)	71 (76.3)	226 (76.6)	0.96	2 (0.5)
Preoperative Creatinine (μmol/l)	89 (73–110)	93.5 (77–125)	87 (72–106)	< 0.01	8 (2.0)
S100B T_0_ (μg/l)	0.27 (0.06–0.89)	0.27 (0.11–0.93)	0.21 (0.05–0.89)	0.41	330 (84.0)

*Patients who died intraoperatively (*n* = 3) were excluded from analysis

Values are presented as *n* (%), mean ± SD or median (interquartile range)

*p*-values represent comparison between neurological injury group and no neurological injury group

*T*_*0*_ Preoperative, *COPD* Chronic obstructive pulmonary disease

Intraoperative data are described in Table 2. Cardiopulmonary bypass time was longer in the neurological injury group compared to the no neurological injury group; 201 min (160–252) versus 189 min (152–227), *p* = 0.04, but there was no significant difference in cross-clamping time or duration of hypothermic circulatory arrest. There were no differences between the groups in terms of arterial cannulation site (femoral cannulation was most common in both groups) or surgical technique used (supracoronary graft landing in ascending aorta was most common in both groups).

**Table 2:** Intraoperative data of the study population

Intraoperative data	All patients *n* = 393	Neurological Injury* *n* = 94	No Neurological Injury* *n* = 298	*p*	Missing
CPB time (min)	191 (155–235)	201 (160–252)	189 (152–227)	0.04	0 (0)
Cross-clamping time (min)	88 (60–130)	92.5 (61–140)	84 (60–128)	0.44	0 (0)
*HCA technique*				0.22	3 (0.8)
Aortic cross-clamp Circulatory arrest ACP RCP	19 (4.9) 193 (49.5) 30 (7.7) 148 (37.9)	3 (3.2) 52 (55.3) 10 (10.6) 29 (30.9)	16 (5.5) 140 (47.8) 20 (6.8) 117 (39.9)		
HCA time (min)	22 (17–29)	22 (16–32)	22 (17–29)	0.78	25 (6.4)
HCA temperature (°C)	18.0 (16.9–20.0)	18.0 (17.0–21.0)	18.0 (16.7–20.0)	0.24	3 (0.8)
*Arterial cannulation site*				0.68	0 (0)
Femoral Axillary Direct aortic Other/Unknown	290 (73.8) 10 (2.5) 83 (21.1) 10 (2.5)	68 (72.3) 2 (2.1) 20 (21.3) 4 (4.3)	220 (74.3) 8 (2.7) 62 (20.9) 6 (2.0)		
*Distal surgical technique*				0.11	0 (0)
Ascending Hemiarch Arch	318 (80.9) 52 (13.2) 23 (5.9)	69 (73.4) 17 (18.1) 8 (8.5)	246 (83.1) 35 (11.8) 15 (5.1)		
*Proximal surgical technique*				0.26	0 (0)
Supracoronary graft Bentall procedure Isolated aortic valve Root replacement/Aortic valve repair replacement	278 (70.7) 85 (21.6) 23 (5.9) 7 (1.8)	70 (74.5) 15 (16.0) 8 (8.5) 1 (1.1)	205 (69.3) 70 (23.6) 15 (5.1) 6 (2.0)		

*Patients who died intraoperatively (*n* = 3) were excluded from analysis

Values are presented as *n* (%) or median (interquartile range)

*p*-values represent comparison between neurological injury group and no neurological injury group

*CPB* Cardiopulmonary bypass, *HCA* Hypothermic circulatory arrest, *ACP* Antegrade cerebral perfusion, *RCP* Retrograde cerebral perfusion

Postoperative data are presented in Table 3. Patients with neurological injury had a larger bleeding during the first 24 h following surgery than patients without neurological injury; 760 ml (550–1370) versus 660 ml (446–900, *p* = 0.01). A higher proportion of patients in the neurological injury group underwent reoperations due to bleeding (21% vs. 13%, p = 0.05) and received significantly more transfusions of red blood cells as well as plasma and platelets compared to the no neurological injury group. Furthermore, patients with neurological injury more often required prolonged ventilatory support (66% vs. 35%, *p* < 0.01). The 30-day mortality as well as in-hospital mortality was significantly higher for patients who suffered neurological injury compared to patients who did not (21.5% . 3.8%, *p* < 0.01) and 23.7% vs. 4.4%, *p* < 0.01, respectively) (Table 4).

**Table 3:** Postoperative data of the study population

Postoperative data	All patients *n* = 395	Neurological Injury* *n* = 94	No Neurological Injury* *n* = 298	*p*	Missing
Recombinant Factor VII	83 (37.9)	23 (43.4)	60 (36.1)	0.34	174 (44.3)
Fibrinogen substitution (g)	4.0 (3.0–8.0)	4.5 (4.0–8.0)	4.0 (3.0–7.0)	0.13	174 (44.3)
Bleeding during first 24 h (ml)	680 (450–940)	760 (550–1370)	660 (446–900)	0.01	178 (45.3)
Reoperation due to bleeding	58 (14.9)	20 (21.3)	38 (12.8)	0.05	3 (0.8)
Red blood cell units	4 (2–8)	5 (2–12)	4 (2–7)	< 0.01	56 (14.2)
Plasma units	4 (0–6)	4 (1–9)	4 (0–6)	0.05	56 (14.2)
Platelet units	4 (2–6)	4 (2–6)	4 (2–4)	< 0.01	56 (14.2)
Ventilation > 48 h	163 (41.9)	61 (65.6)	102 (34.5)	< 0.01	4 (1.0)
Renal replacement therapy	39 (10.0)	13 (13.8)	26 (8.8)	0.16	3 (0.8)
Postoperative MI	15 (14.3)	7 (29.2)	8 (9.9)	0.04	288 (73.3)
Multiple organ failure	7 (2.8)	4 (6.6)	3 (1.6)	0.06	145 (36.9)
Postoperative CKMB	30.0 (19.6–62.3)	34.7 (23.0–75.3)	28.75 (18.0–55.0)	0.03	121 (30.8)
Postoperative Creatinine (μmol/l)	121 (90–201)	153 (102–248)	116 (87–180)	< 0.01	5 (1.3)
Intraoperative death	3 (0.8)	0 (0)	0 (0)	N/A	0 (0)
30-day mortality	34 (8.7)	20 (21.5)	11 (3.8)	< 0.01	4 (1.0)
In-hospital mortality	38 (9.7)	22 (23.7)	13 (4.4)	< 0.01	2 (0.5)

*Patients who died intraoperatively (*n* = 3) were excluded from analysis

Values are presented as *n* (%) or median (interquartile range). *p*-values represent comparison between neurological injury group and no neurological injury group

**Table 4:** Neurological outcomes in the study population

Neurological outcomes	All *n* = 393	Neurological Injury* *n* = 94	No Neurological Injury* *n* = 298	*p*	Missing
Neurological injury	94 (23.9)	94 (100)	0 (0)	N/A	3 (0.8)
Postoperative stroke	74 (19.0)	74 (79.6)	0 (0)	< 0.01	4 (1.0)
Postoperative coma	31 (8.0)	31 (33.3)	0 (0)	< 0.01	4 (1.0)
Transient cerebral event	6 (1.5)	0 (0)	6 (2.0)	0.34	4 (1.0)
S100B T_1_ (μg/l)	0.58 (0.34–1.00)	0.68 (0.32–1.28)	0.56 (0.34–0.95)	0.22	94 (23.9)
S100B T_2_ (μg/l)	0.16 (0.10–0.31)	0.29 (0.13–0.73)	0.14 (0.10–0.23)	< 0.01	81 (20.6)
S100B T_3_ (μg/l)	0.16 (0.09–0.32)	0.24 (0.13–0.60)	0.14 (0.09–0.23)	< 0.01	210 (53.4)
S100B T_4_ (μg/l)	0.18 (0.08–0.34)	0.31 (0.17–0.40)	0.11 (0.08–0.27)	< 0.01	317 (80.7)

*Patients who died intraoperatively were excluded from analysis

Values are presented as *n* (%) or median (interquartile range) *p*-values represent comparison between neurological injury group and no neurological injury group

*T*_*1*_ within 12 h postoperatively, *T*_*2*_ 24 h postoperatively, *T*_*3*_ 48 h postoperatively, *T*_*4*_ 72 h postoperatively

**Fig. 2:**
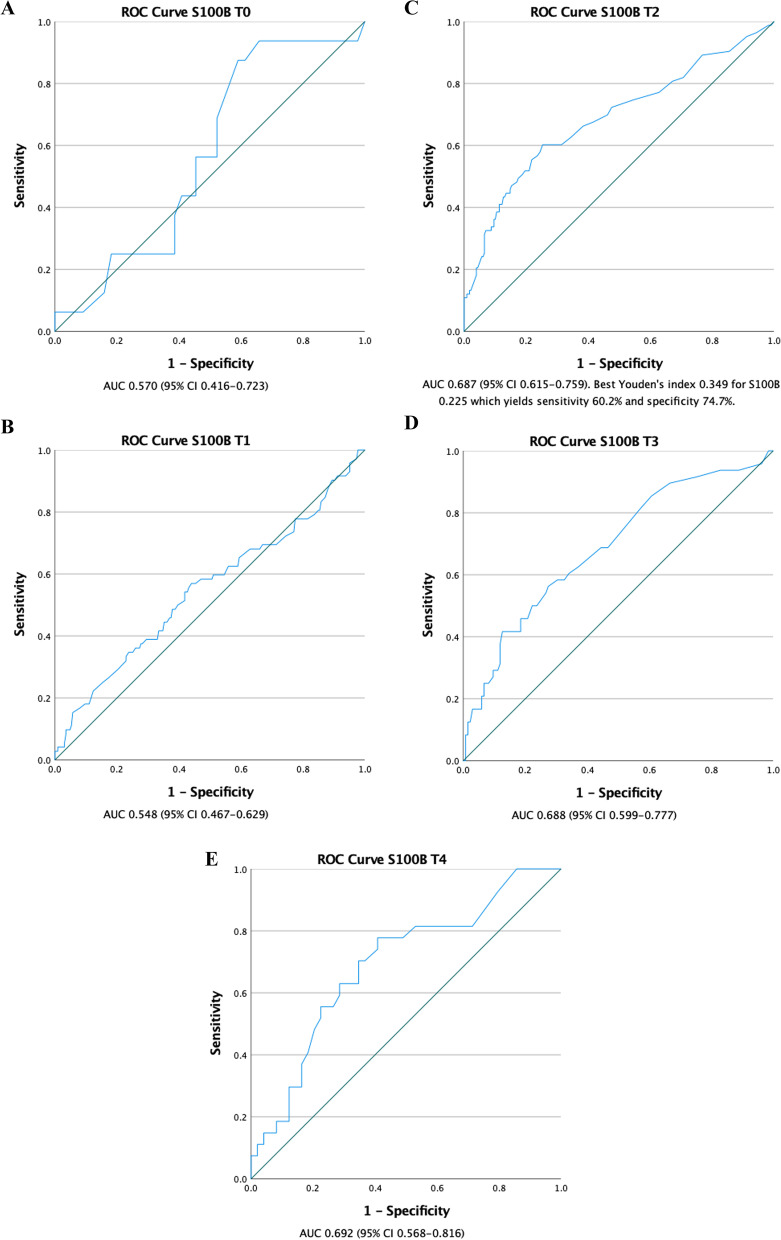
**A** Receiver operating characteristics curve for prediction of neurological injury for S100B at T_0_ (preoperatively). **B** Receiver operating characteristics curve for prediction of neurological injury for S100B at T_1_ (within 12 h postoperatively). **C** Receiver operating characteristics curve for prediction of neurological injury for S100B at T_2._ (24 h postoperatively). **D** Receiver operating characteristics curve for prediction of neurological injury for S100B at T_3._ (48 h postoperatively). **E** Receiver operating characteristics curve for prediction of neurological injury for S100B at T_4._ (72 h postoperatively)

ROC curves with corresponding AUC-values are presented in Figs. 2 A-E. The AUC for the performance of S100B to detect neurological injury was 0.69 for all time points from T2 to T4 (0.687 (95% CI 0.615–0.759), 0.688 (95% CI 0.599–0.777) and 0.692 (95% CI 0.568–0.816) respectively). As we aimed to identify the earliest possible biomarker cutoff to predict neurological injury, we chose to use S100B at T_2_. The best Youden’s index was 0.349 which corresponded to a S100B of 0.225 μg/l and yielded a sensitivity of 60.2% and a specificity of 74.7% for predicting neurological injury.

In Table 5, we present neurological outcomes for all patients with recorded S100B values at T_2_., and a cut-off for S100B of 0.23 μg/l was employed to divide patients into two groups. Neurological injury was more frequent among patients with S100B ≥ 0.23 μg/l compared to patients with S100B < 0.23 μg/l (46% vs. 16% (*p* < 0.01)), as were 30-day mortality and in-hospital mortality (15.7% vs. 3.5% (*p* < 0.01) and 17.6% versus 4.4% (*p* < 0.01), respectively).

**Table 5:** Neurological outcomes–groupwise comparison between S100B T_2_ ≥ 0.23 and S100B T_2_ < 0.23

Neurological outcomes	All *n* = 312	S100B T_2_ ≥ 0.23 *n* = 108	S100B T_2_ < 0.23 *n* = 204	*p*	Missing
Neurological injury	83 (26.6)	50 (46.3)	33 (16.2)	< 0.01	0 (0)
Postoperative stroke	65 (20.9)	35 (32.7)	30 (14.7)	< 0.01	1 (0.)
Postoperative coma	29 (9.3)	24 (22.4)	5 (2.5)	< 0.01	1 (0.3)
Transient cerebral event	5 (1.6)	0 (0)	5 (2.5)	0.17	1 (0.3)
Ventilation > 48 h	141 (45.3)	68 (63.0)	73 (36.0)	< 0.01	1 (0.3)
Renal replacement therapy	34 (10.9)	22 (20.4)	12 (5.9)	< 0.01	0 (0)
Multiple organ failure	6 (3.0)	5 (8.9)	1 (0.7)	< 0.01	111 (35.6)
30-day mortality	24 (7.7)	17 (15.7)	7 (3.5)	< 0.01	2 (0.6)
In-hospital mortality	28 (9.0)	19 (17.6)	9 (4.4)	< 0.01	1 (0.3)

Values are presented as *n* (%)

*p*-values represent comparison between S100B T_2_ ≥ 0.23 group and S100B T_2_ < 0.23 group

*T*_*2*_ 24 h postoperatively

Multivariable logistic regression identified S100B ≥ 0.23 μg/l at T_2_ as an independent predictor for neurological injury (OR 4.71, 95% CI 2.59–8.57; *p* < 0.01, adjusted *p* < 0.01) along with preoperative cerebral malperfusion (OR 4.23, 95% CI 2.03–8.84; *p* < 0.01, adjusted *p* < 0.01) (Table 6). Furthermore, S100B ≥ 0.23 μg/l at T_2_ was identified as an independent predictor for 30-day mortality (OR 4.57, 95% CI 1.18–11.70; *p* < 0.01, adjusted *p* = 0.02) (Table 7). A full presentation of the univariable analyses on both neurological injury and 30-day mortality has been provided (Additional File 1).

**Table 6:** Uni- and multivariable analysis on neurological injury

Outcome on neurological injury	Univariable analysis			Multivariable analysis		
	OR	CI 95%	*p*	OR	CI 95%	*p*
Diabetes Mellitus	1.89	1.10–3.24	0.02	2.48	1.25–4.91	< 0.01
Cerebral malperfusion	3.43	1.92–6.14	< 0.01	4.23	2.03–8.84	< 0.01
CPB time (per 1 min increment)	1.00	1.00–1.01	0.04	1.00	1.00–1.01	0.23
*Proximal surgical technique*						
Supracoronary graft			0.27			0.16
Bentall procedure	0.63	0.34–1.17	0.14	0.42	0.19–0.93	0.03
Isolated aortic valve	1.56	0.64–3.84	0.33	1.03	0.33–3.21	0.96
Root replacement/Aortic valve repair replacement	0.49	0.06–4.13	0.51	0.38	0.04–3.84	0.41
S100B T_2_ (per 0,1 μg/l increment)	1.15	1.07–1.23	< 0.01	1.22	1.01–1.35	< 0.01
S100B T_2_ ≥ 0.23 μg/l	4.47	2.63–7.60	< 0.01	4.71	2.59–8.57	< 0.01

*CPB* Cardiopulmonary bypass, *T*_*2*_ 24 h postoperatively

**Table 7:** Uni- and multivariable analysis on 30-day mortality

Outcome on 30-day mortality	Univariable analysis			Multivariable analysis		
	OR	CI 95%	*p*	OR	CI 95%	*p*
COPD	2.36	0.75–7.36	0.14	3.50	0.95–12.88	0.06
Any malperfusion	3.14	1.48–6.69	< 0.01	3.40	1.37–8.42	0.01
Preoperative Creatinine (per 1 μmol/l increment)	1.01	1.00–1.01	0.04	1.01	1.00–1.02	0.28
Reoperation due to bleeding	3.05	1.35–6.86	< 0.01	2.75	1.03–7.34	0.04
S100B T_2_ ≥ 0.23 μg/l	5.20	2.09–12.99	< 0.01	4.57	1.18–11.70	< 0.01

*COPD* Chronic obstructive pulmonary disease, *T*_*2*_ 24 h postoperatively

## Discussion

To the best of our knowledge, this was the first study to show that elevated S100B 24 h after surgery is a strong independent predictor of neurological injury and 30-day mortality following ATAAD repair. Thus, S100B may serve as a tool for early detection of potential neurological injury following ATAAD surgery.

S100B is a calcium-binding protein primarily found in the glial and Schwann cells of the central nervous system. S100B is not specific to the central nervous system and also can be found in various other tissues including muscles, adipocytes, the heart, and the liver [25]. S100B has a short half-life of about 30–60 min and is mainly eliminated by the kidneys [25]. S100B is released in a biphasic pattern following injury with the first response occurring 3–5 h after the insult[26]. The physiological role of S100B is not yet fully understood but depending on its concentration, it can act as a neurotrophic or a neurotoxic factor with high tissue concentrations driving the latter [25]. Until recent years, S100B has been believed to passively leak from damaged cells and enter the circulation due to the compromised integrity of the blood brain barrier. However, subsequent studies have shown that it is actively released from glial and Schwann cells as a response to stress or damage and that S100B plays an active role in the pathophysiological processes of neurological injury [19].

S100B has been shown to be a reliable marker for cerebral injury in many settings including traumatic brain injury [19] and ischemic as well as hemorrhagic stroke [18]. In the field of cardiac surgery, previous studies have shown that S100B correlates with injured volume and can predict stroke [20]. It has also been shown to be associated with short- and long-term neurobehavioral disorders [21–23]. Johnsson et al*.* for instance, demonstrated that S100B > 0.3 μg/l measured 48 h after cardiac surgery is associated with increased late mortality (follow-up 18 to 42 months) (OR 4.8, 95% CI 2.6–8.8; *p* < 0.001) [27].

ATAAD surgery is associated with high rates of neurological complications caused by both preoperative cerebral malperfusion and the surgical techniques used with hypothermic circulatory arrest [4, 10–15]. Therefore, research and results on S100B in routine cardiac surgery may not be translatable to the complex setting of ATAAD repair. The only available study to investigate S100B after ATAAD surgery included 88 patients, 15 of whom (17%) suffered a stroke after surgery [28]. In that study, the mean S100B concentration 24 h after surgery was similar between the stroke and non-stroke groups (0.31 μg/l vs. 0.29 μg/l (*p* = 0.141)). In contrast to the results of Zhang et al*.,* we found a significant difference in S100B levels between patients with and without neurological injury at 24, 48, and 72 h after surgery, (*p* < 0.01, *p* < 0.01 and* p* < 0.01 respectively). A possible explanation may be that our study population is significantly larger and thus sufficient to demonstrate statistically significant differences between the groups. The study by Zhang et al*.* identifies neurofilament light chain protein (NFL) as a potential predictor of neurological injury with an AUC 12 h after surgery of 0.834 (95% CI 0.723–0.951 p < 0.001). There are, however, disadvantages to NFL: It is not yet readily available for clinical use, and normal cut-off values are not clearly defined.

At T_1_ the mean S100B in both groups was at its highest level, and there was no significant difference between the groups. Previous studies have shown, however, that a significant portion of S100B measured in the blood after heart surgery might be of extracerebral origin, presumably from mediastinal fat and other mediastinal tissues [29]. This phenomenon is exaggerated by the use of cardiotomy suction and autotransfusion [25]. Given the relatively short half-life of S100B, it has been suggested that measurements 24–48 h after surgery would be less influenced by contamination from the surgical site [25]. Therefore, we believe that the results at T_1_ were driven by contamination rather than cerebral injury.

We performed a post hoc sensitivity analysis to assess whether the association between S100B and 30-day mortality maybe an effect of other factors than neurological injury. Once neurological injury was introduced in the multivariable analysis S100B ≥ 0.23 μg/l at T_2_ was no longer significantly associated with 30-day mortality suggesting that neurological injury is a major contributing factor to the predictive ability of S100B for 30-day mortality.

Our results may have important clinical implications. Recent randomized studies have shown favorable outcome for selected stroke patients undergoing thrombectomy compared to medical therapy alone after up to 24 h symptom duration [30, 31]. This further emphasizes the importance of early detection of patients at risk for neurological injury. In the ATAAD-setting, it is difficult to assess patients for neurological injury in the early postoperative phase as they are sedated and require mechanical ventilatory support. It is well known that intrahospital transport of ICU patients is associated with increased risk, including pulmonary complications, hemodynamic alterations and nosocomial infections [32]. With this in mind, it is not only clinically and logistically challenging to obtain radiologic examinations on patients who have undergone ATAAD repair in the early postoperative period, but it entails risk for the patients.

Furthermore, patients suffering neurological injury may benefit from prolonged sedation or other neuroprotective strategies [16, 17, 33]. Consequently, it is important to swiftly identify which patients can benefit from such management. Our study showed that patients with an S100B > 0.23 μg/l 24 h after surgery have an almost fivefold risk of neurological injuries, and that almost half of patients (46%) with S100B > 0.23 μg/l have neurological injuries.

This study is limited by its retrospective study design and the lack of complete series of S100B (values for each time point). However, our analyses showed that there was no difference in the frequency of patients with neurological injury between patients with recorded S100B and those without. Another limitation is the fact that the biomarker analyses have been performed using point of care assays, which may have varied during the study period. In addition, we have only used clinical neurological injury as our primary outcome. It is well known that both routine cardiac surgery and aortic surgery are associated with subclinical neurological lesions on MRI [34, 35]. Furthermore, because the study material spanned more than 20 years, clinical routines have varied and S100B was not recorded for 146 patients. Neurological injury was slightly less common in patients who lacked recorded S100B-values (19% vs. 24%) but there was no significant difference when compared to the included patients. Nevertheless, owing to our large study population and the completeness of follow-up and data collection, we have been able to demonstrate the usefulness of S100B.

## Conclusions

In this study, we demonstrated that S100B 24 h after surgery is a strong independent predictor for neurological injury and 30-day mortality after ATAAD repair. Postoperative S100B may serve as a tool for early detection of neurological injury and aid clinicians in the postoperative management of ATAAD patients.

## Supplementary Information

**Additional file 1. **

## Data Availability

The datasets generated and/or analyzed during the current study are not publicly available due to limitations in the ethical approval but are available from the corresponding author on reasonable request.

## Abbreviations

ATAAD: Acute type A aortic dissection
ROC: Receiver operating characteristic
AUC: Area under the curve
CI: Confidence interval
OR: Odds ratio
HCA: Hypothermic circulatory arrest
IQR: Interquartile range
SD: Standard deviation
VIF: Variance inflation factor
NFL: Neurofilament light chain protein
ICU: Intensive care unit
MRI: Magnetic resonance imaging
