# Supplementary material for: S100B predicts neurological injury and 30-day mortality following surgery for acute type A aortic dissection: an observational cohort study
Source: J Cardiothorac Surg. 2023 Feb 6;18:62. doi: 10.1186/s13019-023-02151-2 (PMC9900954; doi:10.1186/s13019-023-02151-2)
Supplement: Supplementary file 1 — Additional file 1. [file 13019_2023_2151_MOESM1_ESM.docx]

Supplementary table 1. Uni- and multivariable analysis on neurological injury

| OUTCOME ON NEUROLOGICAL INJURY | Univariable analysis | | | Multivariable analysis | | |
| --- | --- | --- | --- | --- | --- | --- |
|  | OR | CI 95% | *p* | OR | CI 95% | p |
| Age (per 1 year increment) | 1.01 | 0.99-1.03 | 0.26 |  |  |  |
| Female | 0.75 | 0.45-1.24 | 0.26 |  |  |  |
| Hypertension | 1.28 | 0.80-2.04 | 0.30 |  |  |  |
| Diabetes Mellitus | 1.89 | 1.10-3.24 | 0.02 | 2.48 | 1.25-4.91 | <0.01 |
| COPD | 0.58 | 0.20-1.74 | 0.33 |  |  |  |
| Smoking | 1.31 | 0.80-2.14 | 0.29 |  |  |  |
| Coronary artery disease | 0.76 | 0.28-2.11 | 0.60 |  |  |  |
| Known thoracic aneurysm | 0.67 | 0.28-1.59 | 0.36 |  |  |  |
| Marfan syndrome | 0.69 | 0.23-2.08 | 0.51 |  |  |  |
| Other connective tissue disease | 0.74 | 0.08-6.67 | 0.74 |  |  |  |
| Family history of dissection | 0.72 | 0.20-2.58 | 0.61 |  |  |  |
| Previous cardiac surgery | 1.26 | 0.24-6.61 | 0.78 |  |  |  |
| Previous aortic surgery | 0.62 | 0.13-2.88 | 0.54 |  |  |  |
| Syncope | 1.78 | 0.98-3.24 | 0.06 |  |  |  |
| Hypotensive shock | 1.60 | 0.92-2.78 | 0.01 |  |  |  |
| Preoperative cardiac arrest | 1.83 | 0.65-5.19 | 0.26 |  |  |  |
| Cardiac tamponade | 1.98 | 1.04-3.76 | 0.04 |  |  |  |
| Any malperfusion | 1.94 | 1.21-3.12 | <0.01 |  |  |  |
| Cerebral malperfusion | 3.43 | 1.92-6.14 | <0.01 | 4.23 | 2.03-8.84 | <0.01 |
| Carotid dissection  None  Unilateral  Bilateral | 1.54  1.47 | 0.83-2.87  0.85-2.55 | 0.26  0.17  0.17 |  |  |  |
| Intramural hematoma | 0.68 | 0.33-1.43 | 0.31 |  |  |  |
| Debakey type 1 | 0.99 | 0.57-1.71 | 0.96 |  |  |  |
| Preoparetive Creatinine (per 1 μmol/l increment) | 1.01 | 1.00-1.01 | <0.01 |  |  |  |
| CPB time (per 1 min increment) | 1.00 | 1.00-1.01 | 0.04 | 1.00 | 1.00-1.01 | 0.23 |
| Clamping time (per 1 min increment) | 1.00 | 1.00-1.01 | 0.51 |  |  |  |
| HCA technique  Aortic cross-clamp  Circulatory arrest  ACP  RCP | 1.98  2.67  1.32 | 0.55-7.08  0.63-11.35  0.36-4.84 | 0.22  0.29  0.18  0.67 |  |  |  |
| HCA time (per 1 min increment) | 1.01 | 0.99-1.03 | 0.38 |  |  |  |
| HCA temperature (per 1 °C increment) | 1.01 | 0.96-1.07 | 0.66 |  |  |  |
| Arterial Cannulation site  Femoral  Axillary  Direct aortic Other/Unknown | 0.81  1.04  2.16 | 0.17-3.90  0.59-1.85  0.59-7.87 | 0.69  0.79  0.88  0.24 |  |  |  |
| Distal surgical technique  Ascending  Hemiarch  Arch | 1.73  1.90 | 0.92-3.28  0.77-4.67 | 0.12  0.09  0.16 |  |  |  |
| Proximal surgical technique  Supracoronary graft  Bentall procedure  Isolated aortic valve  Root replacement/Aortic valve repair  replacement | 0.63  1.56  0.49 | 0.34-1.17  0.64-3.84  0.06-4.13 | 0.27  0.14  0.33  0.51 | 0.42  1.03  0.38 | 0.19-0.93  0.33-3.21  0.04-3.84 | 0.16  0.03  0.96  0.41 |
| S100B T_2_ (per 0,1 μg/l increment) | 1.15 | 1.07-1.23 | <0.01 | 1.22 | 1.01-1.35 | <0.01 |
| S100B T_2_ ≥ 0.23 μg/l | 4.47 | 2.63-7.60 | <0.01 | 4.71 | 2.59-8.57 | <0.01 |

COPD: Chronic obstructive pulmonary disease, CPB: Cardiopulmonary bypass, HCA: Hypothermic circulatory arrest, ACP: Antegrade cerebral perfusion, RCP: Retrograde cerebral perfusion, T_2_ = 24 hours postoperatively.

Supplementary table 2. Uni- and multivariable analysis on 30-day mortality.

| OUTCOME ON 30-DAY MORTALITY | Univariable analysis | | | Multivariable analysis | | |
| --- | --- | --- | --- | --- | --- | --- |
|  | OR | CI 95% | *p* | OR | CI 95% | *p* |
| Age (per year increment) | 1.03 | 1.00-1.07 | 0.07 |  |  |  |
| Female | 0.89 | 0.41-1.94 | 0.77 |  |  |  |
| Hypertension | 1.44 | 0.68-3.05 | 0.34 |  |  |  |
| Diabetes Mellitus | 1.97 | 0.89-4.38 | 0.10 |  |  |  |
| COPD | 2.36 | 0.75-7.36 | 0.14 | 3.50 | 0.95-12.88 | 0.06 |
| Smoking | 1.03 | 0.46-2.31 | 0.94 |  |  |  |
| Coronary artery disease | 1.00 | 0.22-4.51 | 1.00 |  |  |  |
| Known thoracic aneurysm | 0.60 | 0.14-2.64 | 0.50 |  |  |  |
| Marfan syndrome | 0.53 | 0.07-4.08 | 0.54 |  |  |  |
| Other connective tissue disease | N/A | N/A | N/A |  |  |  |
| Family history of dissection | 1.68 | 0.36-7.75 | 0.51 |  |  |  |
| Previous cardiac surgery | 1.93 | 0.23-16.59 | 0.55 |  |  |  |
| Previous aortic surgery | N/A | N/A | N/A |  |  |  |
| Syncope | 2.40 | 1.03-5.60 | 0.04 |  |  |  |
| Hypotensive shock | 1.07 | 0.44-2.63 | 0.88 |  |  |  |
| Preoperative cardiac arrest | 2.73 | 0.73-10.23 | 0.14 |  |  |  |
| Cardiac tamponade | 3.28 | 1.39-7.76 | <0.01 |  |  |  |
| Any malperfusion | 3.14 | 1.48-6.69 | <0.01 | 3.40 | 1.37-8.42 | 0.01 |
| Cerebral malperfusion | 4.34 | 1.97-9.53 | <0.01 |  |  |  |
| Carotid dissection  None  Unilateral  Bilateral | 1.64  0.93 | 0.65-4.13  0.36-2.42 | 0.50  0.30  0.89 |  |  |  |
| Intramural hematoma | 0.41 | 0.09-1.77 | 0.23 |  |  |  |
| Debakey type 1 | 0.88 | 0.38-2.05 | 0.77 |  |  |  |
| Preoperative Creatinine (per 1 μmol/l increment) | 1.01 | 1.00-1.01 | 0.04 | 1.01 | 1.00-1.02 | 0.28 |
| S100B T_0_ (per 1 μg/l increment) | 0.10 | 0.00-5.01 | 0.25 |  |  |  |
| CPB time (per 1 min increment) | 1.00 | 1.00-1.01 | 0.04 |  |  |  |
| Clamping time (per 1 min increment) | 1.00 | 0.99-1.01 | 0.94 |  |  |  |
| HCA technique  Aortic cross-clamp  Circulatory arrest  Antegrade perfusion  Retrograde perfusion | 0.85  0.61  0.63 | 0.18-3.97  0.08-4.72  0.13-3.10 | 0.86  0.83  0.63  0.57 |  |  |  |
| HCA time (per 1 min increment) | 1.01 | 0.98-1.04 | 0.51 |  |  |  |
| HCA temperature (per 1 °C increment) | 1.04 | 0.96-1.13 | 0.29 |  |  |  |
| Arterial cannulation  site  Femoral  Axillary  Direct aortic  Other/Unknown | 1.75  2.48  3.94 | 0.21-14.64  1.11-5.53  0.78-20.02 | 0.09  0.61  0.03  0.10 |  |  |  |
| Distal surgical technique  Ascending  Hemiarch  Arch | 0.75  2.53 | 0.22-2.59  0.80-8.03 | 0.24  0.65  0.12 |  |  |  |
| Proximal surgical technique  Supracoronary graft  Bentall procedure  Isolated aortic valve  Root replacement/Aortic valve repair  replacement | 0.37  1.48  N/A | 0.11-1.24  0.41-5.34  N/A | 0.36  0.11  0.55  N/A |  |  |  |
| Recombinant factor VII | 1.10 | 0.38-3.20 | 0.87 |  |  |  |
| Fibrinogen substitution (per 1 g increment) | 1.21 | 1.07-1.38 | <0.01 |  |  |  |
| Bleeding during first 24 hours (per 1 ml increment) | 1.00 | 1.00-1.00 | <0.01 |  |  |  |
| Reoperation due to bleedning | 3.05 | 1.35-6.86 | <0.01 | 2.75 | 1.03-7.34 | 0.04 |
| Red blood cell units (per 1 unit increment) | 1.01 | 0.99-1.03 | 0.29 |  |  |  |
| Plasma units (per 1 unit increment) | 1.03 | 0.99-1.06 | 0.13 |  |  |  |
| Platelet units (per 1 unit increment) | 1.10 | 1.00-1.21 | 0.06 |  |  |  |
| Ventilation >48h | 6.60 | 2.64-16.50 | <0.01 |  |  |  |
| Renal replacement therapy | 1.40 | 0.46-4.24 | 0.55 |  |  |  |
| Postoperative MI | 2.59 | 0.45-14.74 | 0.29 |  |  |  |
| Multiple organ failure | 23.08 | 4.67-114.07 | <0.01 |  |  |  |
| S100B T_2_ ≥ 0.23 μg/l | 5.20 | 2.09-12.99 | <0.01 | 4.57 | 1.18-11.70 | <0.01 |

COPD: Chronic obstructive pulmonary disease, CPB: Cardiopulmonary bypass, HCA: Hypothermic circulatory arrest, ACP: Antegrade cerebral perfusion, RCP: Retrograde cerebral perfusion, T_2_ = 24 hours postoperatively.
